# The epidemiology and associated factors of non-exclusive breastfeeding: a comparative cross-sectional study of livelihood-secure and insecure areas

**DOI:** 10.3389/fnut.2024.1347780

**Published:** 2024-05-17

**Authors:** Ayenew Negesse, Tsinuel Girma, Beruk Berhanu Desalegn, Marko Kerac, Melkamu Berhane

**Affiliations:** ^1^Academic center of Excellence in Human Nutrition, School of Nutrition, Food Science and Technology (SNFST), Hawassa University, Hawassa, Ethiopia; ^2^Department of Human Nutrition, College of Medicine and Health Science, Debre Markos University, Debre Markos, Ethiopia; ^3^Department of Paediatrics and Child Health, Jimma University, Jimma, Ethiopia; ^4^University of British Colombia (UBC), Vancouver, BC, Canada; ^5^London School of Hygiene and Tropical Medicine (LSHTM), London, United Kingdom

**Keywords:** breastfeeding, Deder, Jimma, Ethiopia, livelihood security

## Abstract

**Introduction:**

Regardless of national commitment, non-exclusive breastfeeding (NEBF) is a public health problem that worsens over time. It can be associated with sociodemographic, economic, and environmental factors and may vary depending on livelihood security. Hence, this study aimed to determine the magnitude of NEBF and identify its associated factors by considering two areas with varied degrees of livelihood security.

**Methods:**

This study represented a comparative cross-sectional survey of 1,060 under 6 months (u6m) infant–mother pairs. Both descriptive and analytic statistics were evaluated using STATA version 17 packages. A binary logistic regression was used to identify associated factors of NEBF. The odds ratio (OR) with a 95% confidence interval (CI) was used to measure the significance of the association at a *p*-value of <0.05.

**Results:**

The pooled magnitude of 51% of NEBF mothers (95% CI: 48.0, 54.0) was 53.1% (95% CI: 49.2, 57.0) and 48.1% (95% CI: 43.4, 52.8) in livelihood-secure and livelihood-insecure areas, respectively. The lack of recollecting the infant’s birth date by mothers (AOR = 2.4; 95% CI = 1.15–4.40) had the highest odds of NEBF while household heads with tertiary education (AOR = 0.14; 95% CI = 0.01–0.54) and the poorest households (AOR = 0.43; 95%CI = 0.20–0.82) had the lowest odds of NEBF in livelihood-secure areas but not in livelihood-insecure areas. Moreover, mothers with male infants (AOR = 1.9; 95% CI = 1.18–2.92) had high odds of NEBF in livelihood-insecure areas but not in livelihood-secure areas. Infants of 2 to less than 4-month-old (AOR = 8.5; 95% CI = 3.47–18.63) and 4 to less than 6-month-old (AOR = 22.2; 95% CI = 8.02–51.97) in livelihood-secure areas and infants of 2 to less than 4-month-old (AOR = 4.3; 95% CI = 1.29–11.67) and 4 to less than 6-month-old (AOR = 8.3; 95% CI = 2.44–22.39) in livelihood-insecure areas had high odds of NEBF.

**Conclusion:**

Over half of the mothers were practicing NEBF, which represents a failure to meet national and international targets. Area vulnerability to livelihood security modifies factors of NEBF. Male infants in insecure areas, infants of unknown age in secure areas, and infants aged 2 months or older, regardless of setting, were more vulnerable to NEBF. However, households with the lowest wealth and higher household head educational status in livelihood-secure areas were less vulnerable to NEBF. Hence, livelihood-based interventions targeting mothers of 2 to less than 6-month-old infants, with emphasis on these factors, may help address and reduce NEBF.

## Introduction

The ideal diet for the optimal health and development of infants aged under 6 months (u6m) is exclusive breastfeeding. Non-exclusive breastfeeding (NEBF) is defined as the practice of introducing solid, semisolid, or liquid foods and/or anything else per mouth to breastfed infants before they reach the age of 6 months (1). Clinically indicated products are however allowed, e.g., oral rehydration solution (ORS) and vitamin and mineral drops or syrups; other medicines are given orally (1).

The World Health Organization (WHO) reported that more than two-thirds of mothers with u6m infants were found to practice NEBF in the years 2015–2020 (2). Other recent studies also show that the frequency of NEBF remains unacceptably high, with large geographic differences (3). It is particularly prevalent in high-income countries, where formula feeding promotion and insufficient maternity leave are among several factors undermining the recommended exclusive breastfeeding (EBF) practices (4, 5). Moreover, in high-income countries, there is also less societal consensus about the importance of promoting EBF practices than in most low- and middle-income countries (LMICs) (3, 6).

Overall, NEBF has slowly declined over the last decade, indicating that it remains a challenge and lagging behind in meeting both national and international targets of reducing by at least 50% and below (3, 7), as well as failing to achieve very good NEBF reduction rates of 0–10% (8).

Unless NEBF is tackled accordingly, it causes multiple problems including anemia due to iron-deficiency (9), infant growth faltering, and associated risk of early childhood mortality (2, 10). There are also large associated economic losses, particularly in sub-Saharan African countries (SSAs) (11). Recent evidence indicates that taking “4 months of age” as an optimal window for starting additional foods against the WHO recommendations at “6 months of age” (1), in both developed and developing countries (12, 13) along with other socioeconomic and demographic factors (14–18), is strongly believed to be associated with a higher magnitude of NEBF.

In Ethiopia, previous studies reported that NEBF prevalence was 28.3% in 2011 in Eastern Ethiopia (17), 47.5% in 2014 in north Ethiopia (19), 39.8% in 2019 in northwest Ethiopia (14), and 49.4% in 2015 in southern Ethiopia (18). Those studies, however, were conducted either in towns or only in rural areas, as well as over a quite longer period of time, which may raise issues of generalizability for the current population, as both the study setting and study period may have a significant effect on the magnitude and associated factors of NEBF (14, 17–19). More importantly, a thorough understanding of NEBF magnitude and its associated factors remains uncertain if studies fail to adequately account for the varying degrees of livelihood security across different population groups. This is because the associated factors of NEBF can be modified depending on the varying degree of livelihood security.

Mothers in relatively livelihood-secure areas with stable economies may have improved maternal nutrition, which is crucial for adequate breastmilk production and a reduced risk of NEBF. However, this may also increase accessibility to complementary foods, including formula (20, 21), and may increase the risk of NEBF (22). Conversely, mothers in livelihood-insecure areas may be unable to buy infant formula or other complementary foods, leaving EBF as their only alternative. These mothers, however, may experience poverty and labor-intensive jobs, resulting in maternal malnutrition and decreased milk production, potentially reinforcing NEBF.

Ethiopia is known among the resource-limited settings with a relatively varied degree of vulnerability to livelihood security across the different areas, for instance, in our study areas, Jimma (23–26) and Deder (27–29).

Jimma is a highland area with a tropical monsoon climate and is noted for its long wet with March to October rainy season, which makes the environment for surplus food crops and coffee production, making the area less vulnerable to livelihood insecurity in Oromia region, southwest Ethiopia (23–26, 30–33). In Jimma, studies reported that formula feeding is a common practice for u6m infants (21) and the level of care provided rather than wealth was reported as a significant predictor of malnutrition (26).

Deder in East Hararghe, a khat-dominant growing area from the Ethiopian lowlands in eastern Ethiopia, has long been known for its vulnerability in terms of climate shock with limited rainfall that exposed the area to repeated drought attacks, population migration, and water scarcity, making this an intervention target area for both the regional administration and other development partners (23–42). The presence of such internally displaced people (IDP) and frequent droughts in Deder have exacerbated livelihood insecurity, poor water, and sanitation practices that may affect infant feeding practices negatively or positively (29, 31–33).

Our recent qualitative study explored that mothers from Jimma areas had a strong habit of initiating complementary feeding at 4 months of age and were strongly defending “EBF until 6 months of infants age is a lie,” which is against the 6 months of recommended practices of EBF (1), whereas most of the mothers and community members from Deder areas discussed and explored that they had a good EBF culture (43).

Therefore, after analyzing differences between the two study sites in terms of sociodemographic, economic, maternal, infant, and other environmental characteristics using formative data from our clinical trial study (44), as well as gaps from our qualitative findings in the two study sites (43), we hypothesized that the epidemiology and factors of NEBF could differ across the aforementioned variables in those comparative areas. Hence, we aimed to conduct a separate and deeper analysis to quantify whether area vulnerability to livelihood insecurity status modifies the association between NEBF and aforementioned variables by examining our formative data preceding a clinical trial (44), which was set in the Jimma and Deder areas.^1^

By doing so, we hope to gain a better and more detailed understanding of the magnitude of NEBF across the different maternal, infant, sociodemographic, economic, and environmental characteristics, as well as its associated factors in two different settings of Ethiopia. This could help policymakers and programmers in contextualizing and strengthening breastfeeding guidelines and policies accordingly.

**Figure 1:**
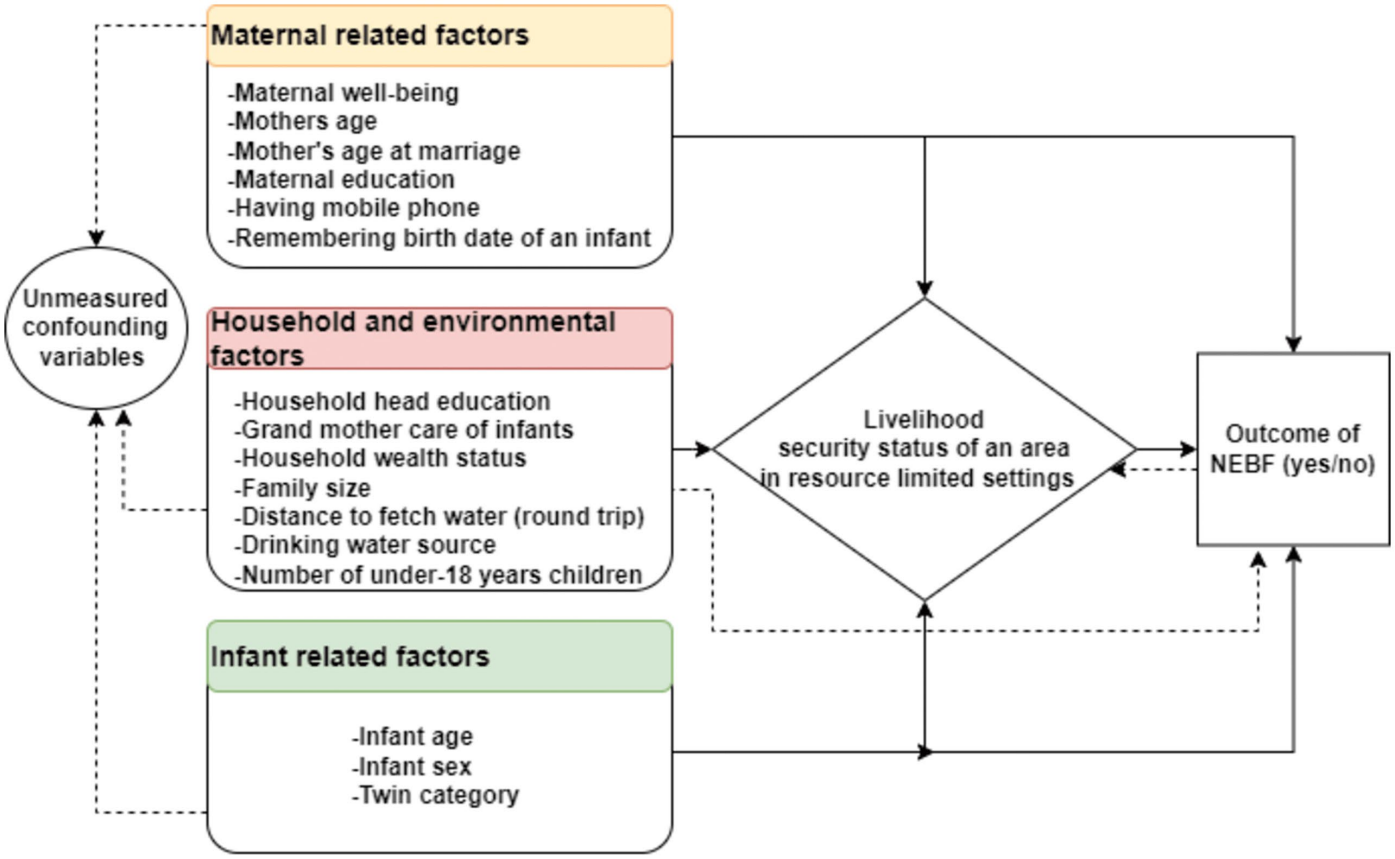
Conceptual framework of factors associated with non-exclusive breastfeeding practice in relatively livelihood-secure areas and insecure areas (adapted from different studies (3, 5, 17, 19, 22, 45)).

Below is a conceptual framework derived from various literature sources, illustrating the hypothetical association between maternal, infant, sociodemographic, economic, and environmental factors and NEBF outcomes with an effect modifier of “area vulnerability to livelihood-insecurity” (Figure 1).

## Methods and materials

### Study design, areas, and period

We present a secondary analysis of previously reported comparative cross-sectional data from selected health centers both from Jimma and Deder, Oromia, Ethiopia (44). Jimma is located 360 km southwest of Addis Ababa, Ethiopia’s capital city, whereas Deder is located 450 km southeast of Addis Ababa. Both Jimma and Deder are densely populated areas having diverse ethnicities, cultural traditions, and religious perspectives, with more than a fourth-fifth of the population being Oromo by ethnicity and Muslim religion followers (46–48).

Jimma zone is characterized by a highland with monsoon tropical climate features, a long annual wet, and almost year round rainy season in southwest Ethiopia (23–26, 30–33), whereas the Deder district is characterized by a tropical lowland climate with high temperature variability, limited rainfall, and known for its repeated drought attacks, a high rate of internally displaced peoples (IDP), and population migration in East Hararghe, eastern Ethiopia (23–27, 30–33, 38–42).

Such variation in our study sites help to gain representative formative data for our clinical trial study, which aims to provide comprehensive critical outcome and implementation evidence to inform national; specifically for Ethiopia regardless of area, cultural, religious, and ethnic diversity as well as for international policy and service development with a view to future sustainable scale (49).

In Jimma, Jimma University (JU) and Jimma University Clinical and Nutrition Research Center (JUCAN) are well known for performing an extensive track record of community-related activities including delivering clinical services, teaching, and diverse research projects. In Deder, GOAL Ethiopia is a well-known partner working on at-risk u6m infant–mother pairs to improve their nutrition and health outcomes. Data were collected between 12 October 2020 and 29 January 2021 (44).

### Population, sample size, and sampling procedure

The study contained the formative cross-sectional survey data from our trial study, which included 1,060 u6m infant–mother pairs (623 were from Jimma and 437 were from Deder) who visited health centers for delivery services, immunization, growth monitoring, and under-five clinics for the treatment of various acute diseases. As a result of their differences in terms of livelihood security and accessibility, we stratified and purposefully selected the Jimma and Deder areas of Ethiopia to obtain complete and representative population groups for our baseline and trial study. Our trial study is a new clinical care pathway (CP) designed for the management of small and nutritionally vulnerable infants and their mothers (MAMI) (50). This was created in order to translate high-level policy guidelines (such as the World Health Organization’s 2013 guidelines on severe malnutrition) into effective front-line clinical and patient management practices (50).

Before using formative data from our trial study, we evaluated sample adequacy by reviewing NEBF and its associated factors reported in a previous study conducted in Ethiopia by Tadesse et al. (18). First, a single population formula was used for checking sample adequacy by considering 49.4% of NEBF practice (18), marginal error = 0.05, the standard normal deviate = 1.96 at 95% CI, design effect = 2, and 10% non-response rate, resulting in a total sample of 847 u6m infant–mother pairs. Second, the double population proportion formula for the sample size estimation technique was considered by undertaking the most commonly cited factor of NEBF practice, in general, and in this study, as “infant age” (18). With 80% power, 10.29 odds of NEBF among 4–5 months old infants compared with 0–1-month-old infants, 22.7% of mothers with 0–1-month-old infants practiced NEBF, a design effect of 2, and a non-response rate of 10%, the maximum sample size calculated using the Fleiss formula with the continuity correction was 75 u6m infant–mother pairs (51). However, the sample estimated using both methods was by far less than our formative data (44). As a result, this study used our MAMI health center survey formative data (44) to determine the magnitude and associated factors of NEBF using livelihood-security status as an effect modifier.

**Figure 2:**
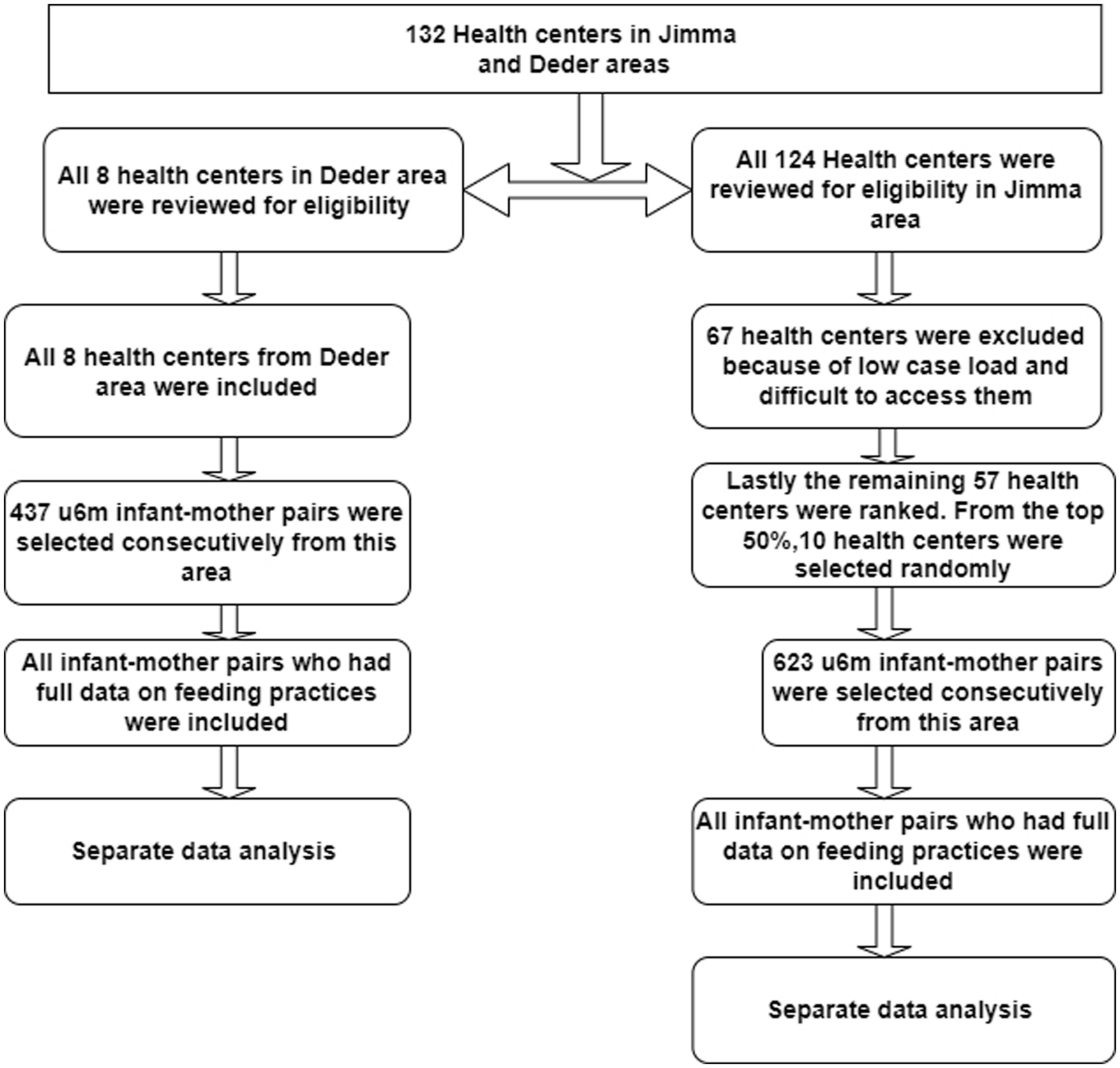
Schematic presentation of the sampling procedures to select the infant–mother pairs.

The original survey sampling procedure was carried out as follows: from the relatively livelihood-insecure areas, we included all eight available health centers and the catchment population in our study, but we used a systematic approach in relatively livelihood-secure areas to pick 10 health centers and their catchment population from a total of 124 alternatives. Then, the following steps were undertaken in our selection process. To begin, we did a thorough assessment of all 124 health center registers to obtain eligible information relative to the ease of access and patient load. As a result of the inadequate eligibility data, we excluded 60 health centers. In addition, we excluded seven health centers that had accessibility issues. Finally, we rated the remaining 57 health centers based on patient load and selected 10 health centers and their catchment population at random from the top 50% of the list to get a representative sample for our investigation. The overall sampling procedures are shown in the figure below (see Figure 2).

### Study variables

The dependent variable was NEBF (yes vs. no), and the independent variables were as follows: infant and mother attributes (infant age, infant sex, maternal age at first marriage, maternal age, maternal educational level, twin category, number of children younger than 18 years within the household, maternal well-being, mobile phone use, grandmother care of infants, and ability to remember infant’s birth date) and household attributes (wealth status, family size, household head educational level, household drinking water source, and distance to fetch water).

### Operational definitions

#### Non-exclusive breastfeeding practice

To generate the NEBF variable, WHO and UNICEF Infant and Young Child Feeding (IYCF) practice assessment tools were used (52, 53). It was assessed by asking questions such as, “Does the infant ever breastfeed?” What was the infant given immediately after birth? Is the infant still breastfeeding? Was the infant on any medications, minerals, or liquids prescribed by a healthcare worker? Was the infant fed any liquids, semisolids, or solids in the last 24 h, day or night? An u6m infant was considered in “NEBF” for this study if he/she had ever breastfed but consumed any liquids, semisolids, or solid foods and/or anything else orally other than medicines, vitamins, or minerals.

#### Maternal wellbeing

It was measured by Patient Health Questionaire-9 (PHQ-9). The total PHQ-9 score ranges from 0 to 27. The severity of caregivers’ mental health is classified as follows: 0 (no depressive disorder); 1–4 (minimum depression); 5–9 (mild depression); 10–14 (moderate depression); 15–19 (moderately severe depression); and 20–27 score (severe depression) (54). To meet the assumptions of Pearson chi-square, the study merged the data and treated them as binary variables, with a score of 0 indicating good maternal well-being and a score of 1–27 indicating poor maternal well-being.

#### Wealth index

It is the total value of a household’s natural, physical, and financial assets minus its liabilities (55). Initially, the reliability test was carried out by the variables used in measuring wealth status. The variables were also entered into the principal component analysis, and the wealth index was calculated as a continuous scale of relative wealth. Wealth quintiles were created by assigning a score to each household; the wealth of each family was classified as follows: one as poorest (lowest), two as poorer (second), three as medium (third), four as rich (fourth), and five as richest (fifth).

#### Area vulnerability to livelihood insecurity

The Livelihood Vulnerable Index (LVI) tool is a latent variable commonly used in developing countries, such as Ethiopia, to aggregate and measure vulnerability to livelihood insecurity (27, 30, 38–43, 56, 57). This tool includes questionnaires on climate change and drought, households’ adaptive capacity or livelihood strategies, household assets, population demography including migration profile, shortage of water, and access to food in the area to be studied (56, 57). In a very recent study conducted in Jimma and Deder, the development partners in collaboration with the regional administration and local experts employed both this tool and historical metrological data to assess and clearly delineate local-scale vulnerability for effective area prioritization and project implementation with the goal of reversing and sustaining livelihoods (23–27, 30–33, 38–43, 58). Hence, based on this empirical information, we directly took the Deder area from a tropical lowland climate in East Hararghe, eastern Ethiopia, as more vulnerable to livelihood insecurity (27, 38, 39, 41, 58) and the Jimma area from a highland monsoon tropical climate in southwest Ethiopia as less vulnerable to livelihood insecurity (23–26, 30–33).

### Data collection, quality management, and assurance methods

As published elsewhere (44), twelve nurses from JUCAN supervised by four individuals with master’s degrees in health sciences collected the data. For 2 weeks, all data collectors and supervisors were trained in data collection tools and data entry techniques via the Research Electronic Data Capture (REDCap) tablet-based application. The data collection modules in REDCap were checked for accuracy. On the same day of the data upload, the collected data were meticulously assessed for completeness and clarity before and after being posted to the REDCap server.

### Statistical analysis

Both the descriptive and analytic statistical analyses were performed using STATA version 17 packages (STATA 17, College Station, Texas 77,845 USA). Descriptive data were summarized using frequency tables and percentages. Before fitting into the final model, multicollinearity diagnosis was performed using the variable inflation factor (VIF) to check any significant intercorrelation issues across the independent factors. Then, a binary logistic regression model was used after checking model fitness via the Hosmer–Lemeshow goodness of fit test (59).

First, bivariate analysis was conducted to examine the association between NEBF and each independent variable. Then, variables with a *p*-value of less than 0.25 in the bivariate analysis (60) and deemed important variables that can be associated with NEBF were included for the final different model building of multiple variable logistic regression. Finally, the Akaike Information Criterion (AIC) was used to evaluate the final model, and the best model with the lowest AIC value was chosen. The odds ratio (OR) with a 95% confidence interval (CI) was used to measure and identify the significant factors of NEBF at a p-value of <0.05.

## Results

### Sociodemographic and economic characteristics

This study included 1,060 u6m infant–mother pairs: 437 (41.2%) from relatively livelihood-insecure areas and 623 (58.8%) from relatively livelihood-secure areas. More than half of the infants (54.6% in livelihood-secure areas and 56.8% in livelihood-insecure areas) were male babies, while 91.5% of mothers in livelihood-secure areas and 90.6% of mothers in livelihood-insecure areas were at the age of 19 years and older. More than three-fourths of mothers in 75.4% of livelihood-secure areas and 89.7% in livelihood-insecure areas married for the first time before the age of 19 years (Table 1). According to the Pearson chi-square statistical tests, there were significant differences (a p-value of <0.05) in sociodemographic, economic, environmental, and infant-related characteristics between the livelihood-secure and livelihood-insecure areas (Table 1). This test implies that factors of infant feeding practice in each area are also different, emphasizing the need to do further separate analysis.

**Table 1:** Sociodemographic and economic characteristics.

Variables	Category	Relatively livelihood-secure area	Relatively livelihood-insecure area	*p*-value
		Number (%)	Number (%)	
Infant sex	Male	340(54.6)	248(56.8)	0.4830
	Female	283(45.4)	189(43.2)	
Infant age	0 to <1 month	63(10.1)	26(5.9)	<0.001*
	1 to <2 month	137(22.0)	49(11.2)	
	2 to <4 months	278(44.6)	176(40.3)	
	4 to <6-months	145(23.3)	186(42.6)	
Twin category	Singleton	611(98)	432(98.8)	0.3180
	Non-singleton	12(1.9)	5(1.1)	
Mother age	<19 years	53(8.5)	41(9.4)	0.6220
	≥19 years	570(91.5)	396(90.6)	
Mother’s age at marriage	<19 years	470(75.4)	392(89.7)	<0.001*
	≥19 years	153(24.6)	45(10.3)	
Maternal education	No education	240(38.5)	194(44.4)	<0.001*
	Primary	275(44.1)	210(48.1)	
	Secondary	71(11.4)	25(5.7)	
	Tertiary	37(5.9)	8(1.8)	
Maternal wellbeing	Good	568(91.9)	373(85.9)	0.003*
	Poor	50(8.1)	61(14)	
Remembering the birth date of the infant	Yes	542(87)	336(76.9)	<0.001*
	No	81(13)	101(23.1)	
Household head education	No education	205(36.3)	135(31.3)	0.001*
	Primary	254(45.0)	198(45.8)	
	Secondary	60(10.6)	79(18.3)	
	Tertiary	45(8.0)	20(4.7)	
Family size	<5 family	388(62.3)	239(54.7)	0.013*
	≥5 family	235(37.7)	198(45.3)	
Telephone for communication	Yes	280(44.9)	105(24.0)	<0.001*
	No	343(55.1)	332(76.0)	
Household drinking water source	Improved	556(89.2)	196(44.9)	<0.001*
	Non-improved	67(10.8)	241(55.1)	
Household wealth	Poorest	156(25.0)	56(12.8)	<0.001*
	Poor	92(14.8)	121(27.7)	
	Middle	86(13.8)	126(28.8)	
	Rich	124(19.9)	88(20.1)	
	Richest	165(26.5)	46(10.5)	

*Indicates significant variables across the Jimma and Deder study areas.

### Magnitude of non-exclusive breastfeeding practice

The pooled magnitude of 51% of NEBF mothers (95% CI: 48.0, 54.0) was 53.1% (95% CI: 49.2, 57.0) and 48.1% (95% CI: 43.4, 52.8) in livelihood-secure and livelihood-insecure areas, respectively (*p*-value = 0.104). More than half of NEBF was practiced among mothers with male infants (62.9% in livelihood-secure and 51.1% in livelihood-insecure areas). A high magnitude of NEBF was reported among infants aged 2 to less than 4 months (49.8%) in livelihood-secure areas and infants aged 4 to 6 months (54.3%) in livelihood-insecure areas. More than three-quarters of early married women practiced NEBF (76.1% in livelihood-secure and 90% in livelihood-insecure areas). A high number of women who did not have mobile phone for communication practiced NEBF (58.6% in livelihood-secure and 76.2% in livelihood-secure areas) (Table 2).

**Table 2:** Magnitude of non-exclusive breastfeeding practice.

		Non-exclusive breastfeeding practice			
		Relatively livelihood-secure area		Relatively livelihood-insecure area	
Variables	Category	Yes (%)	No (%)	Yes (%)	No (%)
Infant sex	Male	132 (62.9)	182 (55)	116 (51.1)	158 (54.1)
	Female	78 (37.1)	149 (45)	111 (48.9)	134 (45.9)
Infant age	0 to <1 month	11(3.3)	52(17)	6(2.9)	20(8.8)
	1 to <2 month	46(13.9)	91(31.2)	13(6.2)	36(15.9)
	2 to <4 months	165(49.8)	113(38.7)	77(36.7)	99(43.6)
	4 to <6-months	109(32.9)	36(12.3)	114(54.3)	72(31.7)
Twin category	Singleton	322(97.3)	289(99.0)	208(99.0)	224(98.7)
	Non-singleton	9(2.7)	3(1.0)	2(1.0)	3(1.3)
Mother’s age	<19 years	23(6.9)	30(10.3)	23(11.0)	18(7.9)
	≥ 19 years	308(93.1)	262(89.7)	187(89.0)	209(92.1)
Mother’s age at marriage	<19 years	252(76.1)	218(74.7)	189(90.0)	203(89.4)
	≥ 19 years	79(23.9)	74(25.3)	21(10.0)	24(10.6)
Maternal education	No education	136(41.1)	104(35.6)	96(45.7)	98(43.2)
	Primary	147(44.4)	128(43.8)	94(44.8)	116(51.1)
	Secondary	32(9.7)	39(13.4)	16(7.6)	9(4.0)
	Tertiary	16(4.8)	21(7.2)	4(1.9)	4(1.8)
Maternal wellbeing	Good	179 (85.2)	300 (90.6)	194 (85.5)	268 (91.8)
	Poor	31 (14.8)	31(9.4)	33 (14.5)	24 (8.2)
Remembering the birth date of the infant	Yes	160 (76.2)	276 (83.4)	176 (77.5)	266 (91.1)
	No	50 (23.8)	55 (16.6)	51 (22.5)	26 (8.9)
Household head education	No education	116(39.5)	89(33.0)	60(29.1)	75(33.2)
	Primary	136(46.3)	118(43.7)	93(45.1)	105(46.5)
	Secondary	23(7.8)	37(13.7)	42(20.4)	37(16.4)
	Tertiary	19(6.5)	26(9.6)	11(5.3)	9(4.0)
Family size	<5 family	124 (59)	197 (59.5)	115 (50.7)	191 (65.4)
	≥5 family	86 (41)	134 (40.5)	112 (49.3)	101 (34.6)
Having mobile phone	Yes	137(41.4)	143(49.0)	50(23.8)	55(24.2)
	No	194(58.6)	149(51.0)	160(76.2)	172(75.8)
Grandmother care of infants	No	217(65.6)	206(70.5)	130(61.9)	128(55.5)
	Yes	214(34.4)	86(29.5)	80(38.1)	101(44.5)
Drinking water source	Improved	295(89.1)	261(89.4)	100(47.6)	98(42.3)
	Non-improved	38(10.9)	31(10.6)	110(52.4)	131(57.7)
Fetching water distance	< 30 min	218(85.2)	217(88.9)	81(41.5)	84(39.4)
	≥30 min	38(14.8)	27(11.1)	114(58.5)	129(60.6)
Household wealth status	Poorest	34 (16.2)	75 (22.7)	22 (9.7)	81 (27.7)
	Poor	58 (27.6)	53 (16)	63 (27.8)	39 (13.4)
	Middle	62 (29.5)	45 (13.6)	64 (28.2)	41 (14)
	Rich	35 (16.7)	72 (21.8)	53 (23.3)	52 (17.8)
	Richest	21 (10)	86 (26)	25 (11)	79 (27.1)

### Factors associated with non-exclusive breastfeeding practice

At first, we stratified the data based on areas, distinguishing between relatively livelihood-secure (Jimma) and insecure (Deder) areas. Subsequently, we conducted separate bivariate and multiple variable logistic regression analyses. Before fitting the data into the final model, we diagnosed multicollinearity among the confounding variables. Maternal education with household head education, household wealth index with family size, maternal and household education with recollection of the infant’s birth date, and household wealth index with a mobile phone were assumed to be confounded. We have performed multicollinearity diagnostics across all the independent variables using VIF, and all of them had a VIF value of <10, indicating that no significant multicollinearity issue was found across the independent variables. However, the number of under 18-year-old children was strongly correlated with household family size, excluding them from the final model according to the settled criteria of data analysis in this study. A binary logistic regression model was used after checking model fitness via the Hosmer–Lemeshow goodness of fit test, which showed a *p*-value of 0.23, indicating good model fit (59).

From the final model, using livelihood security as an effect modifier, we have found different associated factors of NEBF in each area: from the relatively livelihood-insecure areas, mothers with male infants (AOR = 1.8; 95% CI = 1.15–4.61) had the highest odds of NEBF than mothers with u6m female infants. However, the sex of an infant was not statistically significant in relatively livelihood-secure areas (AOR = 0.9; 95% CI = 0.58–1.37). In relatively livelihood-secure areas, maternal lack of recollecting the infant’s birth date (AOR = 2.1; 95% CI = 1.10–4.02) had the highest odds of NEBF, while it was not significant in comparatively livelihood-insecure areas (AOR = 0.9; 95% CI = 0.85–1.43). Furthermore, household heads with tertiary education had lower odds of NEBF than household heads with no education (AOR = 0.12; 95% CI = 0.02–0.76), but this was not significantly associated with relatively livelihood-insecure areas (AOR = 2.6; 95% CI = 0.58–11.68). Similarly, mothers from the poorest households in relatively secure areas had lower odds of NEBF than rich households (AOR = 0.41; 95% CI = 0.21–0.85) but not in generally insecure areas (AOR = 2.1; 95% CI = 0.89–4.75). Furthermore, the age of an infant was also associated with NEBF in both reasonably secure and insecure settings. In relatively secure areas, mothers with 2 to less than 4-month-old infants (AOR = 6.7; 95% CI = 2.95–15.25) and 4 to less than 6-month-old infants (AOR = 16.2; 95% CI = 6.62–39.52) and in insecure areas mothers with 2 to less than 4-month-old infants (AOR = 3.2; 95% CI = 1.15–9.11) and 4 to less than 6-month-old infants (AOR = 5.9; 95% CI = 2.10–16.83) had the highest odds of NEBF (Table 3).

**Table 3:** Factors associated with non-exclusive breastfeeding practice.

Variables	Category	Relatively livelihood-secure area	Relatively livelihood-insecure area
		AOR (95% CI)	AOR (95% CI)
Intercept (β0)		0.4 (0.11, 1.4)	0.1 (0.01, 0.32
Sex of infant	Female	1	1
	Male	0.9 (0.58, 1.37)	1.8* (1.15, 4.61)
Infant age	0 to <1 month	1	1
	1 to <2 month	1.8 (0.75, 4.47)	1.4 (0.41, 4.61)
	2 to <4 months	6.7* (2.95, 15.25)	3.2* (1.15, 9.11)
	4 to <6 months	16.2* (6.62, 39.52)	5.9* (2.10, 16.83)
Twin category	Singleton	1	
	Non-singleton	2.0 (0.37, 10.66)	
Maternal age	<19 years	0.6 (0.25, 1.39)	1.7 (0.74, 3.66)
	≥ 19 years	1	1
Maternal age at marriage	<19 years	1.0 (0.60, 1.66)	0.8 (0.38, 1.60)
	≥19 years	1	1
Mother education	No education	1	1
	Primary	1.0 (0.59, 1.52)	0.8 (0.52, 1.36)
	Secondary	0.8 (0.34, 1.99)	4.1 (0.99, 16.97)
	Tertiary	3.4 (0.39, 30.00)	1.5 (0.17, 13.27)
Maternal wellbeing	Normal	1	1
	Abnormal	1.6 (0.75, 3.36)	1.2 (0.65, 2.29)
Remembering the birth date of the infant	Yes	1	1
	No	2.1* (1.10, 4.02)	0.9 (0.50, 1.43)
Having mobile phone	Yes	1	1
	No	1.0 (0.59, 1.55)	1.8 (0.95, 3.48)
Household head education	No education	1	1
	Primary	0.9 (0.56, 1.46)	1.3 (0.81, 2.18)
	Secondary	0.7 (0.32, 1.68)	1.5 (0.76, 2.89)
	Tertiary	0.1* (0.02, 0.76)	2.6 (0.58, 11.68)
Grandmother help in childcare	Yes	1	1
	No	0.8 (0.52, 1.36)	1.5 (0.97, 2.41)
Family size	< 5	1	1
	≥5	1.3 (0.93, 1.80)	0.8 (0.51, 1.28)
Drinking water source	Improved	1	1
	Non-improved	1.0 (0.53, 1.85)	1.2 (0.69, 1.91)
Fetching water distance	< 30 min	1	1
	≥30 min	1.6 (0.84, 3.21)	0.8 (0.51, 1.28)
Household wealth	Poorest	0.4* (0.21, 0.85)	2.1 (0.89, 4.75)
	Poor	0.9 (0.40, 1.80)	1.5 (0.78, 2.72)
	Middle	0.5 (0.25, 1.11)	1.6 (0.87, 2.97)
	Rich	1	1
	Richest	0.6 (0.32, 1.06)	1.4 (0.63, 3.20)

AOR, Adjusted Odds Ratio; CI, Confidence Interval. *Significant variables.

## Discussion

This study found that more than half (51%) of mothers practiced NEBF: 53.1% in livelihood-secure areas and 48.1% in a livelihood-insecure area. In line with the current findings, one study has also shown that three out of five mothers practiced NEBF, with minimal progress over the last 15 years (61). Similarly, NEBF was reported to be 49.4% in southern Ethiopia in 2016 (18) and 47.5% in northern Ethiopia in 2014 (19). However, the overall finding of NEBF in the current study was considerably higher than in a 2013 study in eastern Ethiopia, which was 28.3% (17) and in a 2019 study in northwest Ethiopia, which revealed that NEBF in the area was 39.8% (14). When compared with the last two NEBF reports, our NEBF data suggest that it is a continuing and even potentially an increasing issue of pressing public health concern, similar to other studies conducted on breastfeeding issues established thus far (5, 6, 62). It is possible that the dynamics of exclusive breastfeeding practice are changing as a result of societal changes, particularly in a market-driven environment (63), whereby u6m traditional breastfeeding-focused infant feeding practices are more challenging to maintain in today’s culture (64). This cultural shift from human milk feeding to market driven foods for infants may explain the increased prevalence of NEBF in our study settings too, plus this may reflect wider challenges as cultural pressures elsewhere in Ethiopia also affect the feeding practices of u6m infants and not to be on track of NEBF rate reduction (43, 65, 66), which may be a barrier for Ethiopia not being on track to achieve the desired reduction in NEBF by 2025 (62).

Hence, the current study along with others mentioned here indicated that the NEBF rate remains high and has shown a slow reduction over the past decade, failing to meet the WHO’s 2021 target for a very good NEBF practice reduction rate of 0–10% (8), failing to reach the Ethiopian Health Sector Transformation Plan (HSTP) 2016–2020 target of reducing NEBF to 28% (67), and also alarming one for not being on track toward progress of NEBF reduction rates in Ethiopia by 2025 (62). This implies that policies aimed at promoting EBF practice must be strengthened and critical gaps need to be addressed according to the local contexts for a successful EBF practice and effective health developmental gains (68).

There is not enough evidence of the differences in the associated factors of NEBF across different degrees of livelihood security. This study and a few others indicated (22, 45) that livelihood security modifies the association between some of the sociodemographic and economic variables and NEBF outcomes. Our study found that, in relatively livelihood-secure areas, mothers who failed to remember their infant’s birth date had significantly higher odds of practicing NEBF than mothers who remembered their infant’s birth date. However, this pattern was not found in livelihood-insecure areas. Though there is no supportive evidence in other similar settings, in reality, not knowing the infant’s birth date is often a symptom of a larger issue—a sense of maternal disempowerment and vulnerability that can result in poor feeding choices, non-responsive feeding, and failure to follow guidelines (69–71). Additionally, mothers who fail to remember their infant’s birth date may incorrectly assume that their infant has already reached the age of 6 months, leading to the introduction of additional foods before the recommended age. Another possible explanation is that educated mothers may use vaccination cards to track their infant’s age and birth date, allowing them to feed infants EBF only until infants reach 6 months. However, mothers with the access and capacity to introduce infant feeds may be trying to justify their actions, based on pretending not to recall their infant’s age, but rather by looking at other developmental cues associated with body language or behaviors of maturity to determine when an infant is ready to eat other foods (69). This is a difficult reality that must be addressed by underscoring the importance of empowering mothers with the knowledge and resources needed to make informed decisions about their infant’s feeding options (72).

Mothers from the poorest households had lower odds of practicing NEBF than mothers from wealthier households in relatively livelihood-secure areas, but this difference was not statistically significant in livelihood-insecure areas.

Similarly, a previous study found that in wealthy countries, one out of every five infants did not exclusively breastfeed, whereas in poor countries, this ratio was one out of every 25 infants (73). Several studies, notably those conducted in Ethiopia and China, have revealed that the poorest mothers had the lowest NEBF rates (74, 75). Societal changes over the 21st century have led to substantial increases in NEBF rates in many nations, not only for those with high and increasing wealth (5) but also for some African countries (64), including Ethiopia (43, 65, 66). The lower NEBF rates among the poorest mothers can be because high-income households have better access to complementary foods than the most economically disadvantaged households (5, 63). This access, together with the means to the early introduction of infant feeds and an attempt to balance the challenges of working life for employed mothers, may further explain this finding.

Similar to high-income countries (3, 5, 6), our study found that NEBF is commonly practiced in wealthier households in Ethiopia. This supported that NEBF remains an unaddressed issue in low-income countries as well (64). Therefore, breastfeeding promotion via social mobilization, mass media, legislation, policy, enforcement, counseling, support, lactation management, and other behavior-change strategies that may reduce NEBF should also be included to target better-off households in low-income settings. However, attention should be paid to the fact that the association between high wealth index with NEBF could be an opportunity for providing adequate and diverse complementary feeds during 6–23 months of an infant age (76), which is crucial for infant growth and development (77).

In livelihood-secure areas, households with heads having tertiary education had lower odds of NEBF than non-educated household heads. Though specific evidence is lacking and further investigation is needed, similar studies conducted, regardless of settings, have consistently documented evidence supporting this finding (14, 74, 78–80). This is because household heads with tertiary education may have a better understanding of the benefits of EBF and the consequences of NEBF; thus, they can defend against interferences and pressures from traditional beliefs and misconceptions about NEBF.

The current study demonstrated that male infants had greater odds of NEBF in relatively livelihood-insecure areas, while this sex disparity was not observed in relatively livelihood-secure areas. Though the underlying factors need more investigation, consistent findings have been reported in Ethiopia (81), Angola (82), Malawi (83), and 29 sub-Saharan African countries (84). This difference is because scholars claimed that programs and policies are more focused on girls than boys to break the intergenerational cycle of malnutrition, so boys’ vulnerability to NEBF and malnutrition (85, 86) may have received little attention in nutrition program design or nutrition policy formulation (87, 88). Hence, program staff and policymakers should be aware of boys’ vulnerability both for NEBF and malnutrition. In livelihood-secure and insecure areas, mothers with infants of 2 months and older had higher odds of practicing NEBF which is in-line with reports from Ethiopia and across continents (18, 81, 89–94). As infants grow and approach 6 months of age, mothers tend to become less confident about EBF and are more likely to follow non-exclusive breastfeeding (NEBF). This may be due to a belief that breast milk alone is insufficient to meet their infants’ nutritional and hydration needs (43, 95–98).

Additionally, while EBF is recommended for the first 6 months of an infant’s life, working mothers may have difficulty continuing EBF as their infants grow older and require more frequent feedings (99). Because of these and others, many mothers may supplement breast milk with complementary foods and/or formula, which leads to a decrease in EBF rates, as infants approach 6 months. Furthermore, our previous qualitative findings also found that there is a cultural belief known as “there is no life without water,” indicating cultural pressure to introduce complementary feeds before the age of 6 months (43). Therefore, it is imperative to underpin a comprehensive package of care that integrates breastfeeding guidelines with all the technical efforts that can break the aforementioned social and cultural norms to achieve a successful NEBF reduction.

To the best of the authors’ knowledge, this is the first study that compares two different populations and examines the factors associated with NEBF practice while incorporating area vulnerability to livelihood security as an effect modifier. This is because Jimma from the central highlands in southwest Ethiopia and Deder from the eastern lowlands in eastern Ethiopia are comparatively dissimilar places in terms of topography, climate category, and vulnerability to livelihood insecurity, as their data are already documented elsewhere (23–27, 30–33, 38, 39, 41, 58). It also investigated the same research question for each independent variable and compared the NEBF practice across the two populations. The results showed that there is a marked difference in the associated factors between livelihood-secure and insecure areas and that livelihood security acts as an effect modifier of NEBF practice in resource-limited settings. Another strength of this study is that data were collected following strict procedures along with daily monitoring and evaluation techniques via a Research Electronic Data Capture (REDCap) tablet-based application linked to its central server, both of which were critical for maintaining data quality and assurance throughout the data collection period.

However, the cross-sectional design of this study limits its ability to establish causality, more specifically, the longitudinal effect of livelihood security on NEBF outcomes, as it can only identify associations between variables rather than determine true cause-and-effect relationships (100, 101). Another limitation of our study could be the nature of feeding-related data collection, which could be influenced by social desirability and recall bias. However, the validated version of the WHO’s and UNICEF’s infant and young child feeding (IYCF) assessment tool (52, 53) was utilized to reduce information and recall biases associated with u6m infant feeding practices and maximize the accuracy of the data we have collected to date. There was also a paucity of data that may affect NEBF in this study, such as measures of women’s empowerment, perceived social support, antenatal care, and the quality of EBF advice/support (81, 102, 103). This secondary analysis also missed religious, ethnic, and cultural data, which unfortunately limited us to further explore their association with NEBF in our study sites. In fact, our recent qualitative findings in the same study areas and a few other studies in low- and middle-income countries reported that religious and cultural narratives were explored as major attributes of NEBF (43, 63, 104).

There is a paucity of information on the association between Ethiopian ethnic diversity and u6m infant breastfeeding practices, especially among minority groups, instead, cultural pressure to introduce pre-lacteal feeds, other than breastmilk, within a few days after birth is observed across all ethnicities (105–107). Established literature studies from the United States, China, and Vietnam have shown marked differences in NEBF practices among different race/ethnic groups (108–110), highlighting the need for further investigation in the Ethiopian context.

Because this study focused on reporting only the epidemiology of NEBF and identifying associated factors in two areas with different climatic zones and vulnerability status, it does not definitively establish causal links between the effect of hot climatic zones and the provision of water and other fluids to u6m infants, as established elsewhere (111–113). As a result, we emphasize the careful interpretation of our findings and highlight the importance of further investigation to quantify the impact of climate change and seasonal variation on the population attributable risk (PAR) of NEBF practice in various climatic zones, particularly in resource-limited settings such as Ethiopia.

It is also possible that some infants older than 6 months were included in the study since mothers were not always sure of the exact age. There may also be selection bias since infants attending health centers do not fully reflect all infants in the catchment population.

## Conclusion

Over half of mothers were practicing NEBF, which represents a failure to meet national and international targets. Livelihood security modifies associated factors of NEBF. Male infants in insecure areas, infants of unknown age in secure areas, and infants aged 2 months or older, regardless of setting, were more vulnerable to NEBF. However, households with the lowest wealth and higher household head educational status in livelihood-secure areas were less vulnerable to NEBF. Hence, livelihood-based interventions targeting mothers of 2 to less than 6-month-old infants, with emphasis on these factors, may help address and reduce NEBF.

## Data availability statement

The raw data supporting the conclusions of this article will be made available by the authors, without undue reservation.

## Ethics statement

The studies involving humans were approved by Jimma University Institutional Review Board (JU-IRB) with a reference number (Ref.No) (IHRPGD/478/2020). Furthermore, the second ethical clearance was also taken from the London School of Hygiene and Tropical Medicine (LSHTM) of Observational/Interventions Research Ethics Committee with the Ref.No (LSHTM Ethics Ref: 18022). The third ethical approval was also obtained from the Hawassa University Institutional Review Board (IRB-HU) with the Ref.No. (IRB/033/14). The studies were conducted in accordance with the local legislation and institutional requirements. Written informed consent for participation in this study was provided by the participants’ legal guardians/next of kin.

## Author contributions

AN: Conceptualization, Data curation, Formal analysis, Investigation, Methodology, Project administration, Software, Validation, Writing – original draft, Writing – review & editing. TG: Conceptualization, Data curation, Investigation, Methodology, Project administration, Supervision, Validation, Writing – review & editing. BD: Conceptualization, Data curation, Investigation, Methodology, Supervision, Validation, Writing – review & editing. MK: Conceptualization, Data curation, Investigation, Methodology, Project administration, Supervision, Validation, Writing – review & editing. MB: Conceptualization, Data curation, Investigation, Methodology, Project administration, Supervision, Validation, Writing – review & editing.

## Footnotes

^1^ https://www.isrctn.com/ISRCTN47300347
